# The Year in Pediatric Electrophysiology: 2022

**DOI:** 10.19102/icrm.2023.14018

**Published:** 2023-01-15

**Authors:** Dustin B. Nash, Kathryn K. Collins

**Affiliations:** ^1^Department of Pediatrics, Division of Pediatric Cardiology, Children’s Hospital Colorado, University of Colorado School of Medicine, Aurora, CO, USA

**Keywords:** Cardiovascular implantable electronic device, catheter ablation, congenital heart disease

In this commentary, we seek to review the most relevant studies of arrhythmias and their treatment in pediatric patients and those with congenital heart disease. The past year in particular has seen knowledge advancements in arrhythmia management, cardiovascular implantable electronic devices, and catheter ablation.

## Arrhythmias and associated conditions: risk assessment and management

Atrial fibrillation (AF) in healthy children (so-called “lone AF”) is rare, making it difficult to study. The largest cohort of healthy children and young adults (<21 years of age) to date with documented AF was collected and assessed in a retrospective multicenter study of 13 heart centers published by El Assaad et al.^1^ Predictors of recurrence after the initial episode were a family history of AF in a first-degree relative <50 years of age (odds ratio [OR], 1.9; *P* = .047) and a longer P–R interval in sinus rhythm (OR, 1.1 per 10 ms; *P* = .37). Of note, AF recurrence did not differ among patients who underwent no treatment (39/125, 31%), started daily anti-arrhythmic therapy (24/63, 38%), or underwent ablation therapy (14/53, 26%; *P* = .39). Ablation of accessory pathways or other re-entrant targets was the only intervention that improved freedom from recurrence (*P* = .013).

Catecholaminergic polymorphic ventricular tachycardia (CPVT) is a life-threatening inherited arrhythmia condition presenting with catecholamine-induced ventricular arrhythmias. Stratification tools for the risk of sudden cardiac death (SCD) in children with CPVT do not exist. A multicenter ambispective cohort of pediatric patients with CPVT was reported by Kallas et al.^2^ The patients were categorized by sex, proband status, and whether the onset of symptoms occurred at <10 years of age or later. Among the 133 analyzed patients, the cardiac event rate was 33% (44/133), with a 3% (4/133) mortality rate. Proband status, but not age at onset of symptoms or sex, predicted an earlier onset of cardiac events.

Junctional ectopic tachycardia (JET) is a frequently encountered arrhythmia following congenital heart disease. A recent study by Rochelson et al.^3^ compared the use of amiodarone to newly available intravenous sotalol in a retrospective single-center study of all patients with postoperative JET over a 5-year period. Successful JET control (defined as a decrease in rate to <170 beats/min or conversion to sinus rhythm with persistent control over 24 h) was achieved by amiodarone in 75% of cases and by sotalol in 83% of cases. The JET rate decreased faster with a sotalol bolus as compared to an amiodarone bolus.

For adult patients with hypertrophic cardiomyopathy, maximal left ventricular wall thickness (MLVWT) is considered a risk factor for SCD. A recent study^4^ of a large international cohort (n = 1075) selected from the International Paediatric Hypertrophic Cardiomyopathy Consortium database sought to discern whether there was a relationship between left ventricular hypertrophy and SCD risk. Over a median follow-up period of 4.9 years, 115 patients (10.7%) had an SCD event. When categorized by MLVWT *z* score, the cohort breakdown was <10 in 598 (58.1%), ≥10 to <20 in 334 (31.1%), and ≥20 in 143 (13.3%) patients. Interestingly, the estimated SCD risk at 5 years had a non-linear, inverted U relationship, peaking at a *z* score of +23, suggesting that those with the most severe hypertrophy actually had a lower risk than the group with a *z* score of around 20. Coexisting risk factors had a summative effect on risk.

## Cardiovascular implantable electronic devices

Congenital complete heart block (CCHB), by virtue of being a rare condition, typically requires implantation of a pacemaker before adulthood. Weinreb et al.^5^ published the results of a retrospective cohort analysis of subjects with CCHB from a single-hospital cohort from 1976–2018 aimed at determining the long-term incidence of cardiac morbidity and mortality and identifying the risk factors. A total of 115 subjects were included with a median age at last follow-up of 15.2 years. Eighty-eight of 115 (77%) patients underwent pacemaker implantation during the study period. Of these, from the available follow-up data, 23% reached the composite outcome of cardiac morbidity (heart failure, cardiomyopathy, cardiac resynchronization therapy) and mortality. There was no significant association between age at diagnosis, fetal diagnosis, maternal antibody status, and the composite outcome.

Investigation into what happens among pediatric patients who underwent placement of secondary prevention implantable cardiac defibrillators was completed by Robinson et al.^6^ In their multicenter retrospective analysis of patients <21 years of age without prior cardiac disease who received an implantable cardioverter-defibrillator following sudden cardiac arrest (SCA), 106 patients were included with a median age of 14.7 years. Of these patients, over a median follow-up period of 3 years, 20 (19%) received appropriate shocks and 16 (15%) received inappropriate shocks. Underlying diagnosis was not associated with an increased incidence of appropriate shock, nor was the lack of a definitive diagnosis after SCA.

The outcomes of lead extraction at a single large pediatric/congenital heart center were reported by Pham et al.^7^ Over the course of 11 years, 113 patients underwent transvenous lead extraction of 162 leads. The authors differentiated simple extractions (use of manual traction or stylet only) and complex extractions (use of powered sheath or femoral extraction techniques). The median lead age at the time of extraction was 7.2 years. Successful extraction occurred in 110 (97%) patients and 159 (98%) leads. Complex extraction techniques were needed for 120 of 144 leads (75%). Notably, patient age at lead implantation, not age at extraction, was an important indicator for needing complex extraction. They reported major complications in 5 (4.4%) patients with no mortality but a high incidence of tricuspid valve injury.

## Catheter ablation

A multicenter retrospective cohort study of children with Wolff–Parkinson–White (WPW) syndrome using the Improving Pediatric and Adult Congenital Treatment (IMPACT) registry was completed by Janson et al.^8^ to evaluate the association of patient weight with outcomes of catheter ablation for pediatric WPW syndrome. Of the 4,456 subjects evaluated from 84 centers, 624 (14%) weighed <30 kg. Mean adverse events were rare in the total cohort, although they occurred at a higher incidence rate in the <30-kg cohort (0.3% vs. 0.05% – *P* = .04). Ablation success was significantly greater in the <30-kg cohort (95% vs. 92% – *P* = .009).

Zook et al.^9^ sought to identify which endpoints when using cryoablation for the treatment of atrioventricular nodal re-entrant tachycardia (AVNRT) may be more associated with recurrence in a single-center retrospective review of patients <21 years of age who were undergoing a first-time AVNRT cryoablation. Of the 256 patients with a median follow-up of 1.9 years, 5% (13/256 patients) experienced a recurrence, which occurred at a median of 0.6 years. There was no difference in recurrence based on demographic or ablation characteristics. Residual slow pathway conduction (A–H jump or single echo beats) was not associated with a higher risk of recurrence compared to the rate among those cases with no residual evidence of slow pathway conduction.
